# Supplementary material for: Prioritized and predictive intelligence of things enabled waste management model in smart and sustainable environment
Source: PLoS One. 2022 Aug 11;17(8):e0272383. doi: 10.1371/journal.pone.0272383 (PMC9371262; doi:10.1371/journal.pone.0272383)
Supplement: Attachment — Submitted filename: Response to Reviewers.pdf [file pone.0272383.s002.pdf]

Response to Reviewers

Article ID:

PONE-D-22-03543

Original Article Title:

Prioritized and Predictive Intelligence of Things Enabled Waste Management Model in Smart and Sustainable Environment

Re: Response to reviewers

Dear Editor-in-chief,

Thank you for allowing a resubmission of our manuscript, with an opportunity to address the reviewers' comments. We appreciate the Editor and the reviewers for taking the time to review our manuscript carefully and for providing valuable feedback to help us improve the quality of the document.

We have made every effort to eliminate all the indicated major and minor inconsistencies and do hope that as a result, the quality of our revised manuscript has improved further. The updated parts are highlighted in red color and the point-to-point responses are given below.

Reviewer 1#

- **The authors could summarize the introduction, it is too long in the present form.**

  - The introduction section is completely restructured and made more compact. Only relevant information is retained.

- **I suggest the authors include the following paper in their literature review: "An IoT-based smart cities infrastructure architecture applied to a waste management scenario", Ad Hoc Networks, Volume 87, 2019, Pages 200-208, ISSN 1570-8705.**

  - Authors have discussed this paper in Section 2 and included the suggested paper in the paper as reference [52].

- **Section 3 could be better structured, divided in subsection according to the subjects handled in the text.**

- Authors have divided the section 3 into 2 sub-sections 3.1 and 3.2 in the revised manuscript according to the content.

- **The results are well presented but not so nicely discussed in Section 4. I recommend the authors to elaborate better on the discussion.**

- The results analysis section 4 is revised and more details about the results are included in the revised manuscript.

- **I suggest the authors divide the text in the conclusion in at least 3 paragraphs. Two for the conclusion, and a third, and last one, for discussions on future works.**

- Authors have segregated the conclusion part into 3 separate paragraphs. Future scope forms the last paragraph of conclusion.

Reviewer 2#

- **Numerous solutions to this problem have been proposed in past research. Please explain novelty and contribution of the study. Why this study is important and what academic values it adds to the field?**

- Though many works have been done in context to this domain, yet our research retains novelty and offers numerous benefits. Majority of existing models are not sustainable enough, incurs considerable delays and is not suitable for large scale applications. They fail to handle different heterogeneous wastes. Also, many models are mostly utilized for only data collection purpose, but decision-making ability is absent in those models. Apart from these concerns, majority of existing solutions either deploys an IoT based module or a simple machine learning based application. A hybrid integrated combination of both sensory based IoT nodes and predictive capabilities are rarely implemented in large scale. In comparison of these existing models, our work offers substantive benefits than others in terms of accuracy, scalability, reliability, robustness with least delays. It is more sustainable and can be applied for more diverse locality with accumulation of heterogeneous garbage. Sensory based data collection and storage can be done in an uninterrupted manner. Also, the collected data can be stored in cloud and can be used for taking decision on number of bins used and identifying denser locality with huge waste accumulation. Random forest is used for classification which makes it more accurate, time saving and free from bias. Also, presence of any discrepancies related to smart bins functioning or presence of poisonous substance can be detected and notified to society.

The important benefit of the model is highlighted in section 4.3.

- **The introduction needs to be rewritten more precise and concrete and providing the much better motivation, significance and impact of the paper. Please explain research gap explicitly then propose research questions.**

- The introduction section is completely restructured and made more compact and precise. Only relevant information is retained. The motivation, research gaps are included with clarity and later the contribution of the research work is written.

- **Include the below references to support your claim:**

- **S.Shanmuga Priya, A.Valarmathi, M.Rizwana, L.Mary Gladence, "Enhanced Mutual Authentication System in Mobile Cloud Environments" in International Journal of Engineering and Technology ISSN: 2227-524X, Vol.7 No.3.34 2018,Page.No 192-197.**
- **Mary Gladence, L., Vakula, C.K., Selvan, M.P., Samhita, T.Y.S., "A research on application of human-robot interaction using artifical intelligence" in International Journal of Innovative Technology and Exploring Engineering (IJITEE) ISSN: 2278- 3075, Volume-8, Issue- 9S2, July 2019.**
- **Jinila, Y. Bevish, V. Rajalakshmi, L. Mary Gladence, and V. Maria Anu. "Food Consumption Monitoring and Tracking in Household Using Smart Container." In Proceedings of the Third International Conference on Computational Intelligence and Informatics, pp. 693-700. Springer, Singapore, 2020.**

- Authors have discussed these papers in Section 2 and included them in the revised manuscript as reference [49], [50] and [51].

- **The discussion of Experiment should be written more in-depth, more precise and concrete, such as what questions were resolved? How can the proposed method solve these problems? Then, the advantages (and disadvantages?) of the proposed methods should be discussed.**

- The result analysis is discussed in more detail in the revised manuscript. The benefits of the proposed model are outlined in section 4.3. The solutions offered in this paper can help to resolve research gaps highlighted in the paper.

- **In the conclusion, the practical application field of the proposed methods and the research findings can be described that highlights the contribution of this article.**

- The conclusion is divided into three paragraphs highlighting the main contribution of the research, methodology used in research and the future scope of the research.

Thanks again for your invaluable comments which significantly improved the paper.

Tarek Gaber

The corresponding author
